# *syn*-5,10,15-Tris(dichloro­meth­yl)-5,10,15-trihy­droxy-5*H*-diindeno­[1,2-*a*:1′,2′-*c*]fluorene dichloro­methane 0.82-solvate

**DOI:** 10.1107/S1600536812020703

**Published:** 2012-05-16

**Authors:** Gregory W. Morrison, Frank R. Fronczek, Steven F. Watkins

**Affiliations:** a Department of Chemistry, Louisiana State University, Baton Rouge, LA 70803, USA

## Abstract

The title compound, C_30_H_18_Cl_6_O_3_·0.82CH_2_Cl_2_, consists of a slightly cup-shaped seven-ring truxene nucleus with hy­droxy and dichloro­methyl substituents at stereocenters 5*R/S*, 10*R/S* and 15*R/S*. C—Cl distances are in the range 1.759 (4)–1.783 (3) Å. Solvent channels parallel to the *b* axis appear to be partially occupied by highly disordered dichloro­methane solvent mol­ecules, the contribution of which were removed from the refinement with the SQUEEZE procedure in *PLATON* [Spek (2009 ▶). *Acta Cryst.* D**65**, 148–155]. Only one of the OH groups forms a hydrogen bond, which is inter­molecular to another OH group, forming centrosymmetric dimers in the crystal.

## Related literature

For further details of the synthesis and information on the synthesis of buckybowls, see: Abdourazak *et al.* (1995 ▶). For applications of truxenes, see: Diring & Ziessel (2009 ▶). Similar structures have been reported by De Frutos *et al.* (1999 ▶); Amsharov & Jansen (2007 ▶) and Menard *et al.* (2011 ▶).
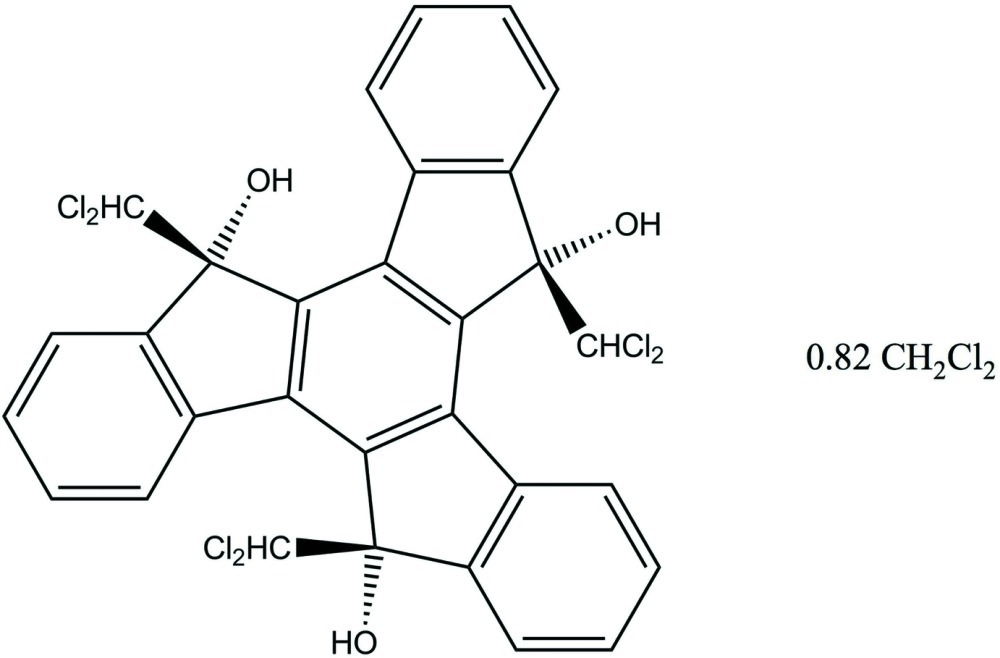


## Experimental

### 

#### Crystal data

C_30_H_18_Cl_6_O_3_·0.82CH_2_Cl_2_
*M* *_r_* = 708.78
Triclinic, 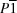

*a* = 10.9719 (4) Å
*b* = 11.6186 (3) Å
*c* = 14.0431 (5) Å
α = 71.009 (2)°
β = 85.291 (2)°
γ = 68.798 (2)°
*V* = 1576.88 (9) Å^3^
*Z* = 2
Mo *K*α radiation
μ = 0.72 mm^−1^
*T* = 90 K
0.38 × 0.13 × 0.05 mm

#### Data collection

Nonius KappaCCD diffractometer with an Oxford Cryostream cooler
Absorption correction: multi-scan (*SCALEPACK*; Otwinowski & Minor, 1997 ▶) *T*_min_ = 0.814, *T*_max_ = 0.972
10851 measured reflections
5744 independent reflections
3608 reflections with *I* > 2σ(*I*)
*R*_int_ = 0.046

#### Refinement

*R*[*F* ^2^ > 2σ(*F* ^2^)] = 0.053
*wR*(*F* ^2^) = 0.156
*S* = 0.97
5744 reflections
356 parameters
H-atom parameters constrained
Δρ_max_ = 0.42 e Å^−3^
Δρ_min_ = −0.32 e Å^−3^

### 

Data collection: *COLLECT* (Bruker, 2004 ▶); cell refinement: *SCALEPACK* (Otwinowski & Minor, 1997 ▶); data reduction: *DENZO* (Otwinowski & Minor, 1997 ▶) and *SCALEPACK*; program(s) used to solve structure: *SHELXS97* (Sheldrick, 2008 ▶); program(s) used to refine structure: *SHELXL97* (Sheldrick, 2008 ▶) and *PLATON* (Spek, 2009 ▶); molecular graphics: *ORTEP-3 for Windows* (Farrugia, 1997 ▶); software used to prepare material for publication: *WinGX* (Farrugia, 1999 ▶).

## Supplementary Material

Crystal structure: contains datablock(s) global, I. DOI: 10.1107/S1600536812020703/kj2201sup1.cif

Structure factors: contains datablock(s) I. DOI: 10.1107/S1600536812020703/kj2201Isup2.hkl

Additional supplementary materials:  crystallographic information; 3D view; checkCIF report

## Figures and Tables

**Table 1:** Hydrogen-bond geometry (Å, °)

*D*—H⋯*A*	*D*—H	H⋯*A*	*D*⋯*A*	*D*—H⋯*A*
O1—H91⋯O3^i^	0.84	2.04	2.834 (3)	158

Symmetry code: (i) 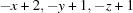.

**Fig. 1.**
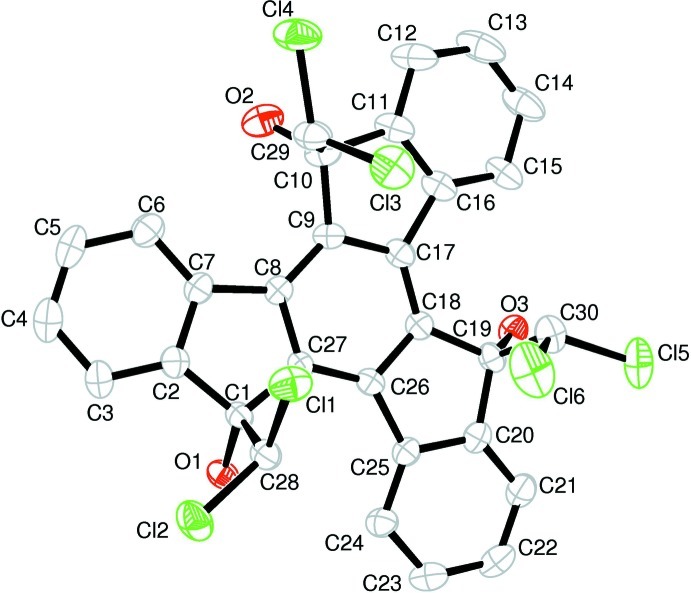
View of the title compound (50% probability displacement ellipsoids). H atoms are not shown.

**Fig. 2.**
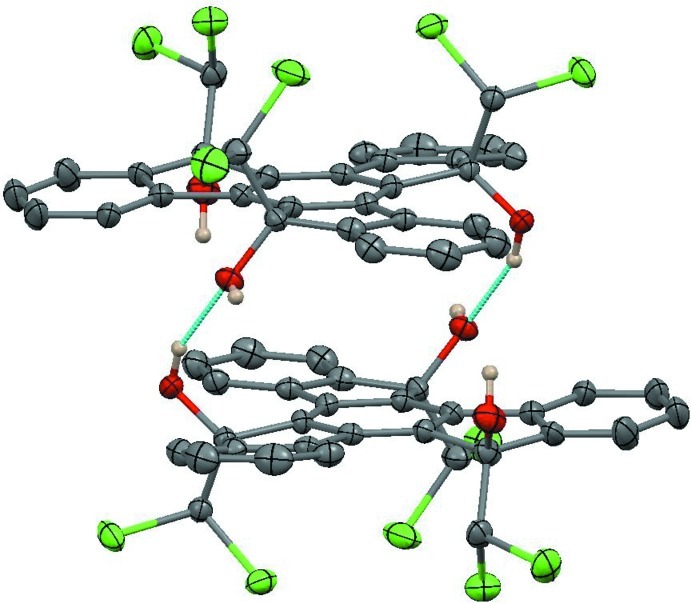
Hydrogen-bonded dimer with 40% probability ellipsoids. Only OH hydrogen atoms are illustrated.

## supplementary crystallographic information

### Comment

The nucleus of the title compound is truxene (C_27_H_18_, CAS: 548–35-6), a nearly planar seven-ring aromatic molecule. Compounds containing this ring system have been previously investigated for use in liquid crystal devices, chiral recognition systems, and fluorescent probes (Diring & Ziessel, 2009). The title compound was synthesized as an intermediate material in the formation of buckybowls (half-buckminsterfullerenes, Abdourazak *et al.*, 1995). Two isomers were separated by chromatography, and the yellow component is herein shown to be the *syn* isomer, with all three OH groups on the same side of the truxene nucleus. The molecule has a slightly cupped shape, with three hydroxy groups oriented toward the inside of the cup and three dichloromethyl groups on the outside of the cup. Relative to the mean plane of the central 6-ring (which is a slightly puckered crown, δ(r.m.s.) = 0.01 (1) Å), the three pairs of carbon atoms on the outer rim of the molecule average 0.36 (1) (C4, C5), 0.15 (1) (C13, C14) and 0.07 (1) (C22, C23) Å above the plane. Of the three OH groups available for hydrogen bond formation, only O1 forms a hydrogen bond, to OH group O3 at 2 - *x*, 1 - *y*, 1 - *z*, thus there are centrosymmetric dimers about 1, 1/2, 1/2, as shown in Fig. 2. A solvent channel with a unit cell volume of 330 Å^3^, parallel to the b axis and centered at 1/2a, displays residual electron density which presumably represents remnants of disordered solvent molecules most of which have evaporated from the crystal since the original synthesis. Procedure SQUEEZE, as implemented in *PLATON* (Spek, 2009), subtracted 69 electrons from the observed structure amplitudes as an approximate solvent contribution.

### Experimental

A solution of lithium dicyclohexylamine was prepared by adding 93 mmol dicyclohexeylamine and 93 mmol n-butyllithium to 75 ml of dried tetrahydrofuran (THF). This solution was added dropwise over one hour to a suspension of 7.3 mmol truxenone in 100 ml of THF and 6 ml of dichloromethane (DCM) at 273 K. The solution was stirred for one hour and then quenched with aqueous ammonium chloride. The THF was removed under reduced pressure and the remaining mixture was extracted with DCM. The resulting organic layer was washed with aqueous citric acid, dried and evaporated. Flash chromatography (silica gel, DCM) was used to isolate 5,10,15-tris(dichloromethyl)-5,10,15-trihydroxy-5*H*-Diindeno[1,2 - a:1',2'-c] fluorene and further chromatography (silica gel, DCM-hexane 3:1) was used to separate the compound into two components. The *syn*- component reported here crystallized from DCM as yellow blades.

### Refinement

Observed structure amplitudes were modified by *PLATON* to eliminate diffuse electron density found in the solvent accessible channel. All H atoms were placed in calculated positions, guided by difference maps, with C—H bond distances 0.95 (aromatic-H) and 1.00 (alkyl-H) Å, O—H distances 0.84 Å, and displacement parameters *U*_iso_=1.2*U*_eq_ (aromatic and alkyl C) and 1.5*U*_eq_ (hydroxyl-O), and thereafter refined as riding. A torsional parameter was refined for each OH group.

### Crystal data

**Table:**

C_30_H_18_Cl_6_O_3_·0.82CH_2_Cl_2_	*Z* = 2
*M**_r_* = 708.78	*F*(000) = 717
Triclinic, *P*1	*D*_x_ = 1.493 Mg m^−^^3^
Hall symbol: -P 1	Mo *K*α radiation, λ = 0.71073 Å
*a* = 10.9719 (4) Å	Cell parameters from 5548 reflections
*b* = 11.6186 (3) Å	θ = 2.6–25.4°
*c* = 14.0431 (5) Å	µ = 0.72 mm^−^^1^
α = 71.009 (2)°	*T* = 90 K
β = 85.291 (2)°	Blade, yellow
γ = 68.798 (2)°	0.38 × 0.13 × 0.05 mm
*V* = 1576.88 (9) Å^3^	

### Data collection

**Table:**

Nonius KappaCCD diffractometer with an Oxford Cryostream cooler	5744 independent reflections
Radiation source: fine-focus sealed tube	3608 reflections with *I* > 2σ(*I*)
Graphite monochromator	*R*_int_ = 0.046
Detector resolution: 9 pixels mm^-1^	θ_max_ = 25.4°, θ_min_ = 2.6°
CCD rotation images, thick slices scans	*h* = −13→13
Absorption correction: multi-scan (*SCALEPACK*; Otwinowski & Minor, 1997)	*k* = −13→13
*T*_min_ = 0.814, *T*_max_ = 0.972	*l* = −16→16
10851 measured reflections	

### Refinement

**Table:**

Refinement on *F*^2^	Secondary atom site location: difference Fourier map
Least-squares matrix: full	Hydrogen site location: inferred from neighbouring sites
*R*[*F*^2^ > 2σ(*F*^2^)] = 0.053	H-atom parameters constrained
*wR*(*F*^2^) = 0.156	*w* = 1/[σ^2^(*F*_o_^2^) + (0.0932*P*)^2^] where *P* = (*F*_o_^2^ + 2*F*_c_^2^)/3
*S* = 0.97	(Δ/σ)_max_ < 0.001
5744 reflections	Δρ_max_ = 0.42 e Å^−^^3^
356 parameters	Δρ_min_ = −0.32 e Å^−^^3^
0 restraints	Extinction correction: *SHELXL97* (Sheldrick, 2008), Fc^*^=kFc[1+0.001xFc^2^λ^3^/sin(2θ)]^-1/4^
0 constraints	Extinction coefficient: 0.0066 (17)
Primary atom site location: structure-invariant direct methods	

### Special details

**Table:**

Geometry. All e.s.d.'s (except the e.s.d. in the dihedral angle between two l.s. planes) are estimated using the full covariance matrix. The cell e.s.d.'s are taken into account individually in the estimation of e.s.d.'s in distances, angles and torsion angles; correlations between e.s.d.'s in cell parameters are only used when they are defined by crystal symmetry. An approximate (isotropic) treatment of cell e.s.d.'s is used for estimating e.s.d.'s involving l.s. planes.
Refinement. Refinement of *F*^2^ against ALL reflections. The weighted *R*-factor *wR* and goodness of fit *S* are based on *F*^2^, conventional *R*-factors *R* are based on *F*, with *F* set to zero for negative *F*^2^. The threshold expression of *F*^2^ > 2σ(*F*^2^) is used only for calculating *R*-factors(gt) *etc*. and is not relevant to the choice of reflections for refinement. *R*-factors based on *F*^2^ are statistically about twice as large as those based on *F*, and *R*- factors based on ALL data will be even larger.

### Fractional atomic coordinates and isotropic or equivalent isotropic displacement parameters (Å^2^)

**Table:**

	*x*	*y*	*z*	*U*_iso_*/*U*_eq_	
C1	0.9165 (3)	0.2835 (3)	0.4130 (2)	0.0263 (8)	
C2	1.0278 (3)	0.2228 (3)	0.3532 (2)	0.0285 (8)	
C3	1.0854 (4)	0.0926 (3)	0.3608 (3)	0.0350 (9)	
H3	1.0534	0.0294	0.405	0.042*	
C4	1.1916 (4)	0.0571 (3)	0.3017 (3)	0.0456 (10)	
H4	1.2323	−0.0319	0.3059	0.055*	
C5	1.2389 (4)	0.1469 (3)	0.2375 (3)	0.0457 (10)	
H5	1.31	0.1198	0.1967	0.055*	
C6	1.1834 (4)	0.2786 (3)	0.2315 (3)	0.0364 (9)	
H6	1.217	0.3409	0.1879	0.044*	
C7	1.0783 (3)	0.3157 (3)	0.2909 (2)	0.0290 (8)	
C8	1.0065 (3)	0.4414 (3)	0.3096 (2)	0.0256 (8)	
C9	1.0110 (3)	0.5655 (3)	0.2661 (2)	0.0287 (8)	
C10	1.0801 (4)	0.6173 (3)	0.1721 (3)	0.0354 (9)	
C11	1.0489 (4)	0.7564 (3)	0.1689 (2)	0.0373 (9)	
C12	1.1007 (5)	0.8464 (4)	0.1053 (3)	0.0513 (11)	
H12	1.1611	0.8243	0.056	0.062*	
C13	1.0610 (5)	0.9699 (4)	0.1163 (3)	0.0573 (12)	
H13	1.0946	1.0329	0.0739	0.069*	
C14	0.9740 (5)	1.0007 (3)	0.1877 (3)	0.0511 (11)	
H14	0.9473	1.0856	0.1934	0.061*	
C15	0.9236 (4)	0.9111 (3)	0.2522 (3)	0.0393 (9)	
H15	0.864	0.9338	0.3018	0.047*	
C16	0.9621 (4)	0.7877 (3)	0.2427 (2)	0.0341 (9)	
C17	0.9350 (3)	0.6691 (3)	0.3023 (2)	0.0290 (8)	
C18	0.8517 (3)	0.6459 (3)	0.3803 (2)	0.0268 (8)	
C19	0.7502 (3)	0.7421 (3)	0.4248 (2)	0.0286 (8)	
C20	0.6942 (3)	0.6552 (3)	0.5068 (2)	0.0306 (8)	
C21	0.6064 (4)	0.6882 (3)	0.5771 (3)	0.0379 (9)	
H21	0.5725	0.7754	0.5784	0.046*	
C22	0.5674 (4)	0.5929 (4)	0.6468 (3)	0.0430 (10)	
H22	0.5065	0.6146	0.6959	0.052*	
C23	0.6178 (4)	0.4662 (4)	0.6442 (3)	0.0409 (9)	
H23	0.5888	0.402	0.6906	0.049*	
C24	0.7099 (4)	0.4311 (3)	0.5749 (2)	0.0328 (8)	
H24	0.7458	0.3434	0.5751	0.039*	
C25	0.7485 (3)	0.5262 (3)	0.5056 (2)	0.0273 (8)	
C26	0.8442 (3)	0.5207 (3)	0.4249 (2)	0.0259 (7)	
C27	0.9198 (3)	0.4203 (3)	0.3888 (2)	0.0250 (7)	
C28	0.7807 (3)	0.2974 (3)	0.3769 (2)	0.0301 (8)	
H28	0.7126	0.3475	0.4147	0.036*	
C29	1.0181 (4)	0.6046 (3)	0.0826 (2)	0.0381 (9)	
H29	1.0298	0.5109	0.0982	0.046*	
C30	0.6479 (4)	0.8437 (3)	0.3406 (3)	0.0382 (9)	
H30	0.6955	0.8851	0.2841	0.046*	
Cl1	0.75059 (9)	0.38564 (9)	0.24653 (6)	0.0407 (3)	
Cl2	0.76348 (10)	0.14417 (8)	0.40087 (7)	0.0441 (3)	
Cl3	0.84936 (10)	0.69516 (9)	0.06527 (7)	0.0513 (3)	
Cl4	1.09646 (12)	0.65136 (9)	−0.03237 (7)	0.0540 (3)	
Cl5	0.53505 (11)	0.96814 (9)	0.38451 (9)	0.0596 (3)	
Cl6	0.56204 (12)	0.76978 (10)	0.29285 (9)	0.0644 (4)	
O1	0.9273 (2)	0.21078 (19)	0.51741 (15)	0.0296 (5)	
H91	1.0026	0.1931	0.5403	0.044*	
O2	1.2147 (3)	0.5482 (3)	0.17035 (19)	0.0452 (7)	
H92	1.2533	0.5455	0.2207	0.068*	
O3	0.8099 (2)	0.8111 (2)	0.46100 (17)	0.0318 (6)	
H93	0.764	0.8384	0.5054	0.048*	

### Atomic displacement parameters (Å^2^)

**Table:**

	*U*^11^	*U*^22^	*U*^33^	*U*^12^	*U*^13^	*U*^23^
C1	0.030 (2)	0.0224 (14)	0.0263 (17)	−0.0106 (14)	0.0010 (15)	−0.0066 (13)
C2	0.026 (2)	0.0287 (16)	0.0279 (18)	−0.0063 (14)	−0.0052 (15)	−0.0080 (14)
C3	0.032 (2)	0.0283 (16)	0.043 (2)	−0.0056 (15)	−0.0052 (18)	−0.0143 (15)
C4	0.039 (2)	0.0364 (19)	0.059 (3)	−0.0018 (18)	−0.004 (2)	−0.0236 (19)
C5	0.033 (2)	0.048 (2)	0.053 (2)	0.0002 (19)	0.001 (2)	−0.029 (2)
C6	0.034 (2)	0.0423 (19)	0.0331 (19)	−0.0113 (17)	0.0019 (17)	−0.0151 (16)
C7	0.027 (2)	0.0331 (16)	0.0288 (18)	−0.0097 (15)	0.0005 (16)	−0.0140 (15)
C8	0.0245 (19)	0.0295 (16)	0.0249 (17)	−0.0098 (14)	−0.0005 (15)	−0.0107 (14)
C9	0.032 (2)	0.0347 (17)	0.0242 (17)	−0.0182 (15)	0.0033 (15)	−0.0093 (14)
C10	0.038 (2)	0.0448 (19)	0.0316 (19)	−0.0234 (18)	0.0078 (17)	−0.0142 (16)
C11	0.057 (3)	0.0422 (19)	0.0252 (18)	−0.0323 (18)	−0.0001 (18)	−0.0098 (16)
C12	0.079 (3)	0.064 (2)	0.032 (2)	−0.050 (2)	0.018 (2)	−0.0173 (19)
C13	0.098 (4)	0.054 (2)	0.039 (2)	−0.056 (3)	0.001 (3)	−0.0072 (19)
C14	0.092 (4)	0.0381 (19)	0.036 (2)	−0.039 (2)	−0.001 (2)	−0.0082 (17)
C15	0.061 (3)	0.0322 (17)	0.0283 (18)	−0.0247 (18)	−0.0066 (18)	−0.0034 (15)
C16	0.044 (2)	0.0349 (17)	0.0269 (18)	−0.0211 (16)	−0.0052 (17)	−0.0050 (15)
C17	0.035 (2)	0.0275 (15)	0.0279 (18)	−0.0148 (15)	−0.0020 (16)	−0.0088 (14)
C18	0.030 (2)	0.0279 (15)	0.0244 (17)	−0.0108 (14)	−0.0003 (15)	−0.0099 (14)
C19	0.031 (2)	0.0244 (15)	0.0323 (18)	−0.0115 (14)	−0.0024 (16)	−0.0094 (14)
C20	0.027 (2)	0.0344 (17)	0.0325 (19)	−0.0086 (15)	−0.0002 (16)	−0.0150 (15)
C21	0.030 (2)	0.0397 (18)	0.047 (2)	−0.0106 (16)	0.0038 (19)	−0.0201 (17)
C22	0.033 (2)	0.059 (2)	0.045 (2)	−0.0178 (19)	0.0121 (19)	−0.0277 (19)
C23	0.042 (2)	0.054 (2)	0.037 (2)	−0.0279 (19)	0.0097 (19)	−0.0162 (18)
C24	0.039 (2)	0.0364 (17)	0.0286 (18)	−0.0171 (16)	0.0061 (17)	−0.0143 (15)
C25	0.027 (2)	0.0311 (16)	0.0253 (17)	−0.0093 (15)	0.0008 (15)	−0.0115 (14)
C26	0.028 (2)	0.0259 (15)	0.0241 (17)	−0.0112 (14)	−0.0016 (15)	−0.0061 (13)
C27	0.0277 (19)	0.0213 (14)	0.0256 (17)	−0.0089 (14)	0.0002 (15)	−0.0066 (13)
C28	0.036 (2)	0.0278 (15)	0.0273 (17)	−0.0127 (15)	0.0015 (16)	−0.0082 (14)
C29	0.052 (3)	0.0415 (18)	0.0271 (18)	−0.0252 (18)	0.0081 (18)	−0.0115 (15)
C30	0.038 (2)	0.0342 (17)	0.042 (2)	−0.0103 (16)	−0.0083 (18)	−0.0120 (16)
Cl1	0.0387 (6)	0.0539 (5)	0.0282 (5)	−0.0186 (4)	−0.0025 (4)	−0.0078 (4)
Cl2	0.0459 (6)	0.0337 (4)	0.0604 (6)	−0.0205 (4)	−0.0039 (5)	−0.0163 (4)
Cl3	0.0539 (7)	0.0569 (6)	0.0443 (6)	−0.0206 (5)	−0.0086 (5)	−0.0141 (5)
Cl4	0.0835 (9)	0.0613 (6)	0.0301 (5)	−0.0419 (6)	0.0162 (5)	−0.0163 (4)
Cl5	0.0493 (7)	0.0383 (5)	0.0795 (8)	0.0047 (5)	−0.0130 (6)	−0.0230 (5)
Cl6	0.0675 (8)	0.0548 (6)	0.0770 (8)	−0.0218 (5)	−0.0331 (6)	−0.0202 (5)
O1	0.0374 (15)	0.0285 (11)	0.0235 (12)	−0.0147 (11)	−0.0026 (11)	−0.0046 (9)
O2	0.0409 (18)	0.0636 (15)	0.0411 (15)	−0.0277 (14)	0.0087 (13)	−0.0209 (13)
O3	0.0332 (15)	0.0343 (12)	0.0341 (13)	−0.0123 (11)	0.0019 (11)	−0.0185 (10)

### Geometric parameters (Å, º)

**Table:**

C1—O1	1.424 (4)	C16—C17	1.481 (5)
C1—C2	1.514 (5)	C17—C18	1.388 (5)
C1—C27	1.526 (4)	C18—C26	1.415 (4)
C1—C28	1.550 (5)	C18—C19	1.523 (4)
C2—C3	1.383 (4)	C19—O3	1.424 (4)
C2—C7	1.406 (5)	C19—C20	1.516 (5)
C3—C4	1.390 (5)	C19—C30	1.550 (5)
C3—H3	0.95	C20—C21	1.369 (5)
C4—C5	1.369 (6)	C20—C25	1.403 (4)
C4—H4	0.95	C21—C22	1.392 (5)
C5—C6	1.403 (5)	C21—H21	0.95
C5—H5	0.95	C22—C23	1.384 (5)
C6—C7	1.389 (5)	C22—H22	0.95
C6—H6	0.95	C23—C24	1.391 (5)
C7—C8	1.485 (4)	C23—H23	0.95
C8—C9	1.388 (4)	C24—C25	1.384 (4)
C8—C27	1.421 (4)	C24—H24	0.95
C9—C17	1.416 (4)	C25—C26	1.481 (4)
C9—C10	1.525 (4)	C26—C27	1.387 (4)
C10—O2	1.406 (4)	C28—Cl1	1.773 (3)
C10—C11	1.512 (5)	C28—Cl2	1.778 (3)
C10—C29	1.549 (5)	C28—H28	1
C11—C16	1.396 (5)	C29—Cl3	1.759 (4)
C11—C12	1.397 (5)	C29—Cl4	1.783 (3)
C12—C13	1.398 (5)	C29—H29	1
C12—H12	0.95	C30—Cl6	1.772 (4)
C13—C14	1.370 (6)	C30—Cl5	1.779 (4)
C13—H13	0.95	C30—H30	1
C14—C15	1.389 (5)	O1—H91	0.84
C14—H14	0.95	O2—H92	0.84
C15—C16	1.389 (4)	O3—H93	0.84
C15—H15	0.95		
			
O1—C1—C2	113.5 (2)	C9—C17—C16	108.6 (3)
O1—C1—C27	115.3 (3)	C17—C18—C26	121.1 (3)
C2—C1—C27	102.3 (3)	C17—C18—C19	129.2 (3)
O1—C1—C28	104.8 (3)	C26—C18—C19	109.5 (3)
C2—C1—C28	113.4 (3)	O3—C19—C20	113.9 (3)
C27—C1—C28	107.7 (2)	O3—C19—C18	110.5 (3)
C3—C2—C7	121.1 (3)	C20—C19—C18	102.7 (2)
C3—C2—C1	127.6 (3)	O3—C19—C30	107.4 (2)
C7—C2—C1	111.0 (3)	C20—C19—C30	113.4 (3)
C2—C3—C4	118.0 (3)	C18—C19—C30	108.8 (3)
C2—C3—H3	121	C21—C20—C25	121.2 (3)
C4—C3—H3	121	C21—C20—C19	128.3 (3)
C5—C4—C3	121.8 (3)	C25—C20—C19	110.5 (3)
C5—C4—H4	119.1	C20—C21—C22	119.3 (3)
C3—C4—H4	119.1	C20—C21—H21	120.3
C4—C5—C6	120.6 (4)	C22—C21—H21	120.3
C4—C5—H5	119.7	C23—C22—C21	119.8 (3)
C6—C5—H5	119.7	C23—C22—H22	120.1
C7—C6—C5	118.5 (3)	C21—C22—H22	120.1
C7—C6—H6	120.8	C22—C23—C24	121.2 (3)
C5—C6—H6	120.8	C22—C23—H23	119.4
C6—C7—C2	120.0 (3)	C24—C23—H23	119.4
C6—C7—C8	132.0 (3)	C25—C24—C23	118.9 (3)
C2—C7—C8	107.9 (3)	C25—C24—H24	120.5
C9—C8—C27	119.1 (3)	C23—C24—H24	120.5
C9—C8—C7	132.6 (3)	C24—C25—C20	119.6 (3)
C27—C8—C7	108.4 (3)	C24—C25—C26	132.1 (3)
C8—C9—C17	121.0 (3)	C20—C25—C26	108.3 (3)
C8—C9—C10	129.1 (3)	C27—C26—C18	119.2 (3)
C17—C9—C10	109.6 (3)	C27—C26—C25	132.1 (3)
O2—C10—C11	113.4 (3)	C18—C26—C25	108.6 (3)
O2—C10—C9	116.3 (3)	C26—C27—C8	120.7 (3)
C11—C10—C9	101.9 (3)	C26—C27—C1	129.5 (3)
O2—C10—C29	104.8 (3)	C8—C27—C1	109.4 (3)
C11—C10—C29	113.9 (3)	C1—C28—Cl1	111.0 (2)
C9—C10—C29	106.7 (3)	C1—C28—Cl2	112.4 (2)
C16—C11—C12	121.1 (3)	Cl1—C28—Cl2	109.33 (18)
C16—C11—C10	111.7 (3)	C1—C28—H28	108
C12—C11—C10	127.2 (3)	Cl1—C28—H28	108
C11—C12—C13	118.1 (4)	Cl2—C28—H28	108
C11—C12—H12	120.9	C10—C29—Cl3	112.3 (2)
C13—C12—H12	120.9	C10—C29—Cl4	112.2 (3)
C14—C13—C12	120.5 (4)	Cl3—C29—Cl4	109.22 (19)
C14—C13—H13	119.8	C10—C29—H29	107.6
C12—C13—H13	119.8	Cl3—C29—H29	107.6
C13—C14—C15	121.7 (3)	Cl4—C29—H29	107.6
C13—C14—H14	119.2	C19—C30—Cl6	111.6 (2)
C15—C14—H14	119.2	C19—C30—Cl5	111.2 (2)
C16—C15—C14	118.7 (4)	Cl6—C30—Cl5	109.7 (2)
C16—C15—H15	120.6	C19—C30—H30	108.1
C14—C15—H15	120.6	Cl6—C30—H30	108.1
C15—C16—C11	119.9 (3)	Cl5—C30—H30	108.1
C15—C16—C17	132.3 (3)	C1—O1—H91	109.5
C11—C16—C17	107.7 (3)	C10—O2—H92	109.5
C18—C17—C9	118.9 (3)	C19—O3—H93	109.5
C18—C17—C16	132.5 (3)		
			
O1—C1—C2—C3	42.4 (5)	C17—C18—C19—C20	−179.8 (3)
C27—C1—C2—C3	167.2 (3)	C26—C18—C19—C20	5.5 (3)
C28—C1—C2—C3	−77.1 (4)	C17—C18—C19—C30	59.8 (4)
O1—C1—C2—C7	−132.3 (3)	C26—C18—C19—C30	−114.9 (3)
C27—C1—C2—C7	−7.4 (3)	O3—C19—C20—C21	54.4 (5)
C28—C1—C2—C7	108.3 (3)	C18—C19—C20—C21	174.0 (3)
C7—C2—C3—C4	−2.6 (5)	C30—C19—C20—C21	−68.8 (5)
C1—C2—C3—C4	−176.8 (3)	O3—C19—C20—C25	−123.5 (3)
C2—C3—C4—C5	0.0 (6)	C18—C19—C20—C25	−3.9 (4)
C3—C4—C5—C6	1.8 (6)	C30—C19—C20—C25	113.3 (3)
C4—C5—C6—C7	−1.0 (6)	C25—C20—C21—C22	−2.0 (5)
C5—C6—C7—C2	−1.5 (5)	C19—C20—C21—C22	−179.7 (3)
C5—C6—C7—C8	173.5 (3)	C20—C21—C22—C23	0.1 (6)
C3—C2—C7—C6	3.4 (5)	C21—C22—C23—C24	1.8 (6)
C1—C2—C7—C6	178.4 (3)	C22—C23—C24—C25	−1.9 (5)
C3—C2—C7—C8	−172.7 (3)	C23—C24—C25—C20	0.0 (5)
C1—C2—C7—C8	2.4 (4)	C23—C24—C25—C26	178.7 (3)
C6—C7—C8—C9	8.3 (6)	C21—C20—C25—C24	2.0 (5)
C2—C7—C8—C9	−176.2 (4)	C19—C20—C25—C24	−179.9 (3)
C6—C7—C8—C27	−171.2 (3)	C21—C20—C25—C26	−177.1 (3)
C2—C7—C8—C27	4.3 (4)	C19—C20—C25—C26	1.0 (4)
C27—C8—C9—C17	2.7 (5)	C17—C18—C26—C27	−1.1 (5)
C7—C8—C9—C17	−176.7 (3)	C19—C18—C26—C27	174.1 (3)
C27—C8—C9—C10	−170.7 (3)	C17—C18—C26—C25	179.6 (3)
C7—C8—C9—C10	9.8 (6)	C19—C18—C26—C25	−5.2 (4)
C8—C9—C10—O2	−54.7 (5)	C24—C25—C26—C27	4.6 (6)
C17—C9—C10—O2	131.2 (3)	C20—C25—C26—C27	−176.5 (3)
C8—C9—C10—C11	−178.6 (3)	C24—C25—C26—C18	−176.3 (4)
C17—C9—C10—C11	7.4 (4)	C20—C25—C26—C18	2.6 (4)
C8—C9—C10—C29	61.8 (4)	C18—C26—C27—C8	1.6 (5)
C17—C9—C10—C29	−112.2 (3)	C25—C26—C27—C8	−179.4 (3)
O2—C10—C11—C16	−131.7 (3)	C18—C26—C27—C1	−170.7 (3)
C9—C10—C11—C16	−6.0 (4)	C25—C26—C27—C1	8.4 (6)
C29—C10—C11—C16	108.5 (3)	C9—C8—C27—C26	−2.4 (5)
O2—C10—C11—C12	45.7 (5)	C7—C8—C27—C26	177.2 (3)
C9—C10—C11—C12	171.4 (4)	C9—C8—C27—C1	171.3 (3)
C29—C10—C11—C12	−74.1 (5)	C7—C8—C27—C1	−9.1 (4)
C16—C11—C12—C13	−1.3 (6)	O1—C1—C27—C26	−53.4 (5)
C10—C11—C12—C13	−178.4 (4)	C2—C1—C27—C26	−177.1 (3)
C11—C12—C13—C14	0.1 (6)	C28—C1—C27—C26	63.2 (4)
C12—C13—C14—C15	0.9 (7)	O1—C1—C27—C8	133.7 (3)
C13—C14—C15—C16	−0.7 (6)	C2—C1—C27—C8	10.0 (3)
C14—C15—C16—C11	−0.5 (5)	C28—C1—C27—C8	−109.8 (3)
C14—C15—C16—C17	175.1 (4)	O1—C1—C28—Cl1	−179.55 (18)
C12—C11—C16—C15	1.5 (6)	C2—C1—C28—Cl1	−55.2 (3)
C10—C11—C16—C15	179.0 (3)	C27—C1—C28—Cl1	57.2 (3)
C12—C11—C16—C17	−175.1 (3)	O1—C1—C28—Cl2	−56.7 (3)
C10—C11—C16—C17	2.4 (4)	C2—C1—C28—Cl2	67.6 (3)
C8—C9—C17—C18	−2.2 (5)	C27—C1—C28—Cl2	−179.9 (2)
C10—C9—C17—C18	172.3 (3)	O2—C10—C29—Cl3	−175.6 (2)
C8—C9—C17—C16	179.0 (3)	C11—C10—C29—Cl3	−51.1 (4)
C10—C9—C17—C16	−6.4 (4)	C9—C10—C29—Cl3	60.5 (3)
C15—C16—C17—C18	8.0 (7)	O2—C10—C29—Cl4	−52.1 (3)
C11—C16—C17—C18	−176.0 (4)	C11—C10—C29—Cl4	72.4 (4)
C15—C16—C17—C9	−173.5 (4)	C9—C10—C29—Cl4	−176.0 (2)
C11—C16—C17—C9	2.5 (4)	O3—C19—C30—Cl6	−177.7 (2)
C9—C17—C18—C26	1.4 (5)	C20—C19—C30—Cl6	−50.9 (3)
C16—C17—C18—C26	179.8 (3)	C18—C19—C30—Cl6	62.7 (3)
C9—C17—C18—C19	−172.7 (3)	O3—C19—C30—Cl5	−54.8 (3)
C16—C17—C18—C19	5.7 (6)	C20—C19—C30—Cl5	71.9 (3)
C17—C18—C19—O3	−57.9 (4)	C18—C19—C30—Cl5	−174.5 (2)
C26—C18—C19—O3	127.4 (3)		

### Hydrogen-bond geometry (Å, º)

**Table:**

*D*—H···*A*	*D*—H	H···*A*	*D*···*A*	*D*—H···*A*
O1—H91···O3^i^	0.84	2.04	2.834 (3)	158

Symmetry code: (i) −*x*+2, −*y*+1, −*z*+1.
